# Circadian Rhythm and Alzheimer’s Disease

**DOI:** 10.3390/medsci6030052

**Published:** 2018-06-21

**Authors:** Jan Homolak, Monika Mudrovčić, Barbara Vukić, Karlo Toljan

**Affiliations:** 1 Department of Pharmacology, University of Zagreb School of Medicine, Šalata 3, 10 000 Zagreb, Croatia; homolakjan@gmail.com; 2 University Hospital for Infectious Diseases, Mirogojska cesta 8, 10 000 Zagreb, Croatia; monikamudrovcic@yahoo.com; 3 University of Zagreb School of Medicine, Šalata 3, 10 000 Zagreb, Croatia; bvukic1@gmail.com; 4 Department of Pathophysiology, University of Zagreb School of Medicine, Kišpaticeva 12, 10 000 Zagreb, Croatia

**Keywords:** Alzheimer’s disease, circadian rhythm, melatonin, pathophysiology, sleep

## Abstract

Alzheimer’s disease (AD) is a neurodegenerative disorder with a growing epidemiological importance characterized by significant disease burden. Sleep-related pathological symptomatology often accompanies AD. The etiology and pathogenesis of disrupted circadian rhythm and AD share common factors, which also opens the perspective of viewing them as a mutually dependent process. This article focuses on the bi-directional relationship between these processes, discussing the pathophysiological links and clinical aspects. Common mechanisms linking both processes include neuroinflammation, neurodegeneration, and circadian rhythm desynchronization. Timely recognition of sleep-specific symptoms as components of AD could lead to an earlier and correct diagnosis with an opportunity of offering treatments at an earlier stage. Likewise, proper sleep hygiene and related treatments ought to be one of the priorities in the management of the patient population affected by AD. This narrative review brings a comprehensive approach to clearly demonstrate the underlying complexities linking AD and circadian rhythm disruption. Most clinical data are based on interventions including melatonin, but larger-scale research is still scarce. Following a pathophysiological reasoning backed by evidence gained from AD models, novel anti-inflammatory treatments and those targeting metabolic alterations in AD might prove useful for normalizing a disrupted circadian rhythm. By restoring it, benefits would be conferred for immunological, metabolic, and behavioral function in an affected individual. On the other hand, a balanced circadian rhythm should provide greater resilience to AD pathogenesis.

## 1. Introduction

Alzheimer’s disease (AD) is a neurodegenerative disorder primarily characterized by deteriorating cognitive functions and neuropsychiatric symptoms [1,2,3]. It is the most prevalent form of dementia, accounting for at least two thirds of cases, whereas vascular dementia is second with a 15% share. Frontotemporal and associative cortical atrophy, ventricular enlargement, and hippocampal volume reduction are macroscopic neuropathological findings appearing in AD [4], although these are not specific or diagnostic per se. Microscopic pathological entities traditionally include accumulation of amyloid β (Aβ), and hyperphosphorylated tau, a microtubule protein. Clinical diagnosis is usually preceded by a longer asymptomatic phase. An estimated 24 million people in the world suffered from AD in 2011 [1], the number being 40 million in 2016 [2], ultimately predicted to reach 131 million in year 2050 [5]. Alzheimer’s disease mostly affects older populations. Increases in incidence have come to a halt in developed countries, whereas trends are still incremental in developing countries [6]. 

Current biomarkers offer tools to rule out AD, but confirming existing disease at an earlier stage would be invaluable for medical practice. At the time of manifest disease when clinical diagnosis is confirmed by specialist neurological and neuropsychological examination, the process of AD pathogenesis has likely been ongoing for prior years [2]. There are multiple available or proposed biomarkers, ranging from cerebrospinal fluid molecule concentrations (e.g., amyloid, tau) to brain morphometry or functional imaging such as computerized tomography (CT), magnetic resonance imaging (MRI), or positron emission tomography (PET) [7]. Currently, large-scale and long-term population data on the progression of the disease with implications for medical management of comorbidities are still unsatisfying and additional quality studies are needed in order to come up with a more effective strategy (while also not neglecting the bioethical specifics of AD) [8,9,10,11]. Based on biomarkers used to track patients predisposed for developing autosomal dominant AD [12], an exemplary undertaking is the Dominantly Inherited Alzheimer Network Trials Unit through which an investigative platform for pharmacological interventions in autosomal dominant AD has been set [13]. Current medical treatments targeting AD primarily consist of cholinesterase inhibitors (donepezil, galantamine, and rivastigmine), and an *N*-methyl-d-aspartate antagonist (memantine), but medications to manage neuropsychiatric symptoms are often added. With multiple attempts to address proposed targets leading to Aβ accumulation, not a single trial demonstrating benefits of a biologic therapy that could be implemented widely has been established [2,14]. Neurosurgical options offering neurostimulation by vagus nerve stimulation or deep brain stimulation are still low-scale experimental [15]. Innovative attempts such as omentum to brain transplant [16], or patient-derived fibroblast gene therapy with subsequent implantation to brain tissue have also been made [16]. An interesting feature of intranasal insulin applied as a possible treatment offers some hope, as the insulin pathway has recently gained much attention regarding etiopathogenesis of AD [17]. Ineffective therapies based strictly on elimination of final end-product and characteristic pathological entity, namely Aβ, prompt the biomedical community to turn to studying the pathophysiological and molecular aspects of AD with a greater focus on underlying complexities, such as neuroinflammation, glial cell activation, and mitochondrial dysfunction [18]. Recent focus on saliency of circadian rhythm has brought an additional component when studying AD pathophysiology, but also when developing or addressing therapeutic targets [19]. This narrative review represents a comprehensive approach to the relationship between circadian rhythm and AD, describing etiopathogenesis and clinical applications.

## 2. Shared Pathogenic Mechanisms of Alzheimer’s Disease and Circadian Rhythm Dysfunction

The cause of Alzheimer’s disease is poorly understood. One of the main risk factors for AD is older age [2]. The strongest genetic risk factors are autosomal dominant genes involved in Aβ processing: presenilin-1 (*PSEN1*), *PSEN2*, and amyloid precursor protein (*APP*), but represent a comparatively small portion (1–5%), of the entire disease prevalence [6,20]. The sporadic form of the disease represents a majority of cases with still unresolved pathophysiology characterized by complex interactions of multiple genetic and environmental factors [21,22]. A gene that potentially increases susceptibility to AD is *ApoEε4*, and 15–20% of AD cases may be associated with it. Some modifiable risk factors have been detected and it seems protection is conferred by higher educational status, social and physical activity, mentally stimulating tasks, light-to-moderate alcohol consumption, and vegetable or fish (omega-3 fatty acids) intake [6,20]. Risk factors increasing the risk for AD are midlife metabolic syndrome, diabetes, smoking, cerebrovascular disease, nutrient deficiencies, traumatic head injuries, and occupational exposure to toxins [6]. Certain risk factors seem to be age-specific. Lowering of blood pressure, body mass index, or cholesterol in older age is associated with higher occurrence of AD [2,6,20].

A number of processes considered to be important for disease pathogenesis are pathophysiologically intertwined with the circadian system, thus representing potentially shared pathogenic mechanisms relevant for AD. However, available data are insufficient to evaluate clinical significance of the theoretical pathophysiological background presented in the text. The main reason for this is the lack of methodologically adequate clinical research and the use of transgenic animals for modelling sporadic form of the disease, which may not be an appropriate model [23].

### 2.1. Amyloid-β Production and Clearance

In 1991, the amyloid cascade hypothesis was proposed to settle the “cause or consequence“ debate that goes back to the time of Alois Alzheimer [21]. Since the discovery of the disease, the central question of the etiopathogenesis of AD was if histopathology drives the disease or if it is just a marker of some other central process, with Dr. Alzheimer personally being fonder of the latter idea [24]. The amyloid cascade hypothesis, the most influential hypothesis favored both by academic research and pharmaceutical industry for the past 20 years, advocates that deposition of amyloid-β peptides in the brain parenchyma is the central event that drives Alzheimer’s disease pathology [14]. Although the relationship of amyloid-β and Alzheimer’s disease has been continually recognized both in humans and in animal models, Aβ deposition is also present in cognitively healthy individuals [25,26,27]. Neurodegeneration can occur without plaque depositions [28]. Adults with Down’s syndrome (with triple copies of *APP*) do not always develop dementia regardless of the presence of diffuse non-fibrillary plaques and elevated Aβ [29]. Strikingly, all of the amyloid-β-centric approaches that reached Phase III clinical trials have failed so far [14]. However, it is still possible that weak or nonexistent correlations between Aβ plaques and dementia may be the result of the extremely complex background of amyloid pathology and possible differences in animal and human pathophysiology [30]. Thus, regardless of its importance in etiopathogenesis of AD, once accumulated, Aβ represents a significant pathophysiological burden and can cause cellular dysfunction.

Current evidence suggests circadian rhythm and the sleep–wake cycle have a role in regulating levels of Aβ. Experimental data shows that both wild type and transgenic Tg2576 mice have a pronounced diurnal rhythm of Aβ as demonstrated by in vivo microdialysis, with peak concentrations of Aβ occurring during wakefulness [31]. Similar diurnal variations were also observed in human cerebrospinal fluid (CSF) samples [31,32,33]. Human CSF Aβ concentrations measured by lumbar catheter over 36 h form a cosine wave consistent with a diurnal pattern demonstrating a 25% difference between highest (during wakefulness) and lowest (during sleep) concentrations [32]. Brain interstitial fluid (ISF) Aβ is positively correlated with time spent awake and negatively correlated with time spent asleep, the amount of non-rapid eye movement (REM) sleep being in most pronounced negative correlation [31]. One of mechanistic links is the recently uncovered variant of lymphatic drainage present in the central nervous system (CNS), which includes a network of genuine lymphatics alongside meningeal vasculature [34] as well as the parenchymal glymphatic system [35]. The latter component, governed by glial cells, might appear as a salient one for elucidating the restorative function of sleep [36,37]. It has been demonstrated that sleep, be it natural or induced by anesthetics, increases the interstitial fluid space by 60% in the brain, thus enabling a greater convective glymphatic flow and clearance of (toxic) metabolites including Aβ [38]. Additionally, central noradrenergic activation is inversely correlated with glymphatic clearance and sleep is a state of markedly reduced noradrenergic stimulation [38,39]. These findings point that glymphatic function is primarily related to the state of arousal and not a circadian clock directly. Notably, total sleep deprivation leads to deleterious effects, causing death in a matter of days or weeks if an experimental animal is subjected to continuous wakefulness [40]. This is also true in humans, as seen in patients suffering from fatal familial insomnia [40]. Moreover, sleep deprivation increases Aβ plaques in transgenic mice [31], and conversely, sleep decreases production and secretion of Aβ and potentiates its clearance [38]. Aged mice have a 40% relative decrease in glymphatic flow, which is concordant with old age being a prominent risk factor for AD [40]. The amplitude of the CSF Aβ cosine wave is most pronounced in healthy adults (aged 18–60 years), decreased in adults older than 60 years negative for amyloid deposition by Pittsburgh compound B (PiB)-PET, and even further decreased in adults older than 60 years with amyloid positive PiB-PET findings [32].

Kang et al. reported that an intracerebroventricular orexin infusion significantly increased ISF Aβ, while the infusion of dual orexin receptor antagonist almorexant decreased ISF Aβ levels and abolished diurnal fluctuations [31]. However, since orexin administration regulates sleep–wake cycle, the question still remains whether the observed effect was in fact a reflection of neuronal activity, otherwise known to be in correlation with Aβ dynamics [41]. On the other hand, a study published by Ma and colleagues indicates that hippocampal orexin signaling can also influence expression of clock-controlled-genes, some of which are directly related to the production of Aβ such as *Bace1* and *Bace2* [42]. Other researchers have also reported on the close relationship of AD related genes and the circadian clock. *PSEN2* gene, one of the regulators of Aβ levels, is rhythmically expressed in suprachiasmatic nuclei (SCN) [13,43,44], and its peripheral tissue expression is controlled by CLOCK:BMAL dimers through both transcriptional and post-translational mechanisms [43].

Chronic sleep deprivation is a recognized risk factor for cardiovascular diseases and immune system alterations [45,46]. In the context of above described glymphatic dysfunction, it is also pathophysiologically implicated in neurodegenerative and neuroinflammatory diseases [47]. There has been data associating sleep disturbances with AD, PD, and multiple sclerosis [48,49,50], but cause-effect relationship is never clear. An interesting finding is that good-quality sleep attenuated the risk for developing AD in *ApoEε4* carriers [51]. In light of experimental data, circadian rhythm disruption that lowers duration and quality of sleep, poses as a self-perpetuating vicious cycle in a bi-directional pathophysiological relationship with the neurodegenerative process [52]. 

### 2.2. Tau Protein Homeostasis

In order to explain the diversity of the events that have been shown to trigger neurodegeneration, the tau hypothesis has been proposed with tau phosphorylation and aggregation as the final common pathway in an integrative model [53,54]. Tau hyperphosphorylation can be triggered by genetic mutations that alter function or isoform expression [54,55], as well as through numerous non-genetic mechanisms such as Aβ oligomers, free radicals, iron overload, neuronal raft cholesterol levels, low density lipoprotein species, homocysteine, and microglial modulation [53]. Triggered tau hyperphosphorylation leads to disassembly of microtubules with consequential sequestration of tau, microtubule associated proteins, and ubiquitin into paired helical filaments (PHF) and neurofibrillary tangles (NFT). Accumulated PHF and NFT damage cytoplasmic function, axonal transport and cellular homeostatic processes [56,57]. It has been shown that tau hyperphosphorylation can also trigger neurodegeneration in other diseases in the absence of Aβ changes [54,57,58], as well as that its presence or phosphorylation and aggregation pattern seem to be in some correlation with the progression of cognitive symptoms [59,60,61]. However, only one tau-directed compound that has so far reached phase III randomized controlled trial (RCT), valproic acid, has had no effect on cognition and functional status [62]. Further elucidation of both the physiological and pathophysiological role of tau protein regulation pathways is needed to truly understand its complex role in AD and direct the development of new specific tau-centric drugs [63].

Sleep deprivation and circadian rhythm dysfunction could lead to dysfunctional tau metabolism [64]. Currently available experimental data is not sufficient for complete understanding of circadian-induced changes of tau. However, there is some insight regarding potential tau-induced changes in circadian rhythm. Tau-related pathological characteristics appear in multiple areas involved in sleep regulation such as locus coeruleus, medial parabrachial nucleus, dorsal raphe nucleus, periaqueductal gray matter, hypothalamic tuberomammilary nucleus, lateral hypothalamus, and basal forebrain [65]. Many of these changes occur during the pretangle stages, indicating potential involvement of tau in sleep and circadian disturbance during the development of the disease. Data from transgenic animals suggest that tau pathology alone can induce sleep disruption associated with neurodegeneration [65,66]. 

### 2.3. Inflammatory Hypothesis

Inflammation triggered in the nervous system is somewhat similar to the peripheral inflammatory cascade, but with certain specifics that are collectively termed neuroinflammation. A great body of literature suggests inflammatory pathways are activated in brains of individuals diagnosed with Alzheimer’s disease [56,67,68]. It is still debated how the inflammation starts and what the main drivers of the inflammatory state are, but it seems that it is maintained through a complex interplay between microglial cells, astrocytes, oligodendrocytes, and neurons [56,67,68]. 

Microglial cells are considered to be the key players in brain inflammatory processes in many diseases [69,70]. Microglial cells can be activated by Aβ, α synuclein, pesticides, viruses, air pollutants, particles from damaged cells, and proinflammatory cytokines [71]. This stimulates the production and release of cytotoxic factors, proinflammatory cytokines, chemokines such as reactive oxygen species (ROS), tumor necrosis factor (TNF)-α, nitric oxide (NO), interleukin (IL)-1β, IL-6, IL-8, macrophage inflammatory protein (MIP)-1α, IL-12, IL-18, and even some enzymes such as insulin degrading enzyme (IDE) [56,71], with a consequent self-perpetuating pro-inflammatory response [72,73,74]. In addition to shared cytokine release with microglia, astrocytes also downregulate the expression of aquaporin-4 channels and significantly reduce glymphatic system clearance [75]. Peripheral stimuli may trigger a central neuroinflammatory response directly via infection or tissue damage and subsequent pathogen-associated molecular protein (PAMP) or damage-associated molecular protein (DAMP) induced immune cascades [67,76]. Amyloid-β in itself is an acute phase response component with anti-microbial potency [77] and is able to activate the complement system [78]. This has prompted investigations on infection-triggered inflammatory process as a causative agent in AD [78]. However, AD is still considered to be a multifactorial disease with no single culprit. 

A positive feedback loop between Aβ and underlying neuroinflammation is a likely pathogenic feature in AD. An indirect neuroinflammatory route is constituted via chronic systemic low grade inflammation that disrupts blood-brain barrier impermeability, cytokine-mediated signalization which activates microglia or summons trafficking immune cells, and neurogenic inflammation mediated by cranial nerves such as the vagal nerve [72,73,74,79]. The latter is currently a topic of special interest in studies focusing on microbiota-gut-brain axis interactions in AD [52,80,81].

A circadian rhythm dictates the profile of the immune system response through individual cellular mechanisms, as well as systemic neurohormonal regulation through the autonomic nervous system and cortisol signaling [82,83,84,85]. During the active phase, leukocyte recruitment is increased, along with circulating levels of epinephrine, norepinephrine and TNF-α, whereas the resting phase is characterized by lower expression of endothelial adhesion molecules and an enhanced pooling of immune cells from the bone marrow to the blood [86]. Disruption of the central ‘zeitgeber’ clock leads to a desynchronized peripheral response and, ultimately, a state of higher immunological vulnerability ensues. Therefore, a connection between bacterial infections and AD [86] could comprise a bi-directional positive feedback loop. The microbiota is also in close relationship with the circadian rhythm, but implications for AD and gut-brain axis could be based on indirect metabolic alterations leading to systemic insulin resistance and inflammation, ultimately increasing the risk for the aforementioned disease [87]. Disruption in any of these components yields a bi-directional response in which a vicious cycle is initiated. Due to growing evidence uniting immune and metabolic features, an immunometabolical perspective is warranted in order to address neuroinflammation comprehensively. The process of ‘inflammaging’ appears as a consequence of accumulated damage from chronic low grade inflammation, insulin resistance, and other aging-associated alterations, including behavioral ones such as disrupted sleep patterns [88,89]. This also speaks in favor of investigating the roles of melatonin and insulin as the components modulating neuroinflammation in AD pathogenesis. Clinical evidence with benefits obtained in patients experimentally treated for AD by anti-inflammatories [68], sleep-modifying therapies [90], or intranasal insulin [17], corroborates the notion.

### 2.4. Oxidative Stress

Most ROS are byproducts of the mitochondrial electron transport chain. If their production exceeds the cellular capacity to remove them, ROS can mediate oxidative damage to DNA, proteins or lipids, and induce cell death. A large body of evidence supports the existence of increased oxidative stress in AD. Low concentrations of the endogenous antioxidants glutathione and catalase together with high oxygen consumption (20–30%) and high content of polyunsaturated fatty acids (PUFAs) make the brain a susceptible target for lipid peroxidation, which damages neuronal membranes and yields several secondary products that disrupt cellular functioning [91,92]. Lipid peroxidation produces highly reactive electrophilic aldehydes such as malondialdehyde (MDA), 4-hydroxy-2-nonenal (HNE), and acrolein, all found to be increased in AD brain [91,93,94]. Lipid peroxidation also seems to be present in subjects with mild cognitive impairment (MCI) and preclinical AD. This makes it pathophysiologically relevant for the pathogenesis of the disease and clinically relevant as a potential biomarker for disease progression and therapeutic efficacy [93,95]. Oxidative stress also damages proteins [96] and nucleic acids [97] with potential implications for the etiopathogenesis and the progression of the disease. 

The etiology of oxidative stress in AD is still debated. It is possible that oxidative stress is generated as a result of the inhibition of the electron transport chain with consequent accumulation of electrons in the complex I and coenzyme Q, where they can be donated directly to molecular oxygen [98]. Another possibility is that it is generated as a consequence of deficient antioxidant systems also observed in AD [56]. A growing body of evidence suggests that circadian regulation of protein expression plays an important role in oxidative stress regulation. In rat cerebral cortex, a day-night cycle of both oxidative damage and the activity of antioxidative enzymes such as glutathione peroxidase and superoxide dismutase was reported back in 1985 [99]. Circadian rhythmicity of various antioxidants and enzymes that protect the cell from oxidative damage has been shown in humans as well. Glutathione peroxidase, glutathione reductase, catalase, superoxide dismutase, uric acid, and peroxiredoxins’ peaks are observed in the morning and melatonin, plasma thiols, and ascorbic acid peak in the evening or during night [100]. The importance of circadian regulation of oxidative stress is further highlighted by findings in knock-out animals [101] suggesting circadian dysregulation could potentiate oxidative damage and encourage development of age-related pathological changes [100].

### 2.5. Vascular Function

The vascular hypothesis, first proposed in 1993, is based on experimental and clinical data pointing to persistent chronic brain hypoperfusion as a common denominator of the harmful effects of all known AD risk factors and the ultimate factor which causes neurodegeneration [102]. Rat models of chronic brain hypoperfusion (the main model used for understanding the progressive pathological changes discussed in the vascular hypothesis) reveal changes that seem pathophysiologically more closely related to AD than vascular dementia. Regions most susceptible to hypoperfusion induced changes are hippocampal CA1 and posterior parietal cortices [102,103]. After the initial hippocampal blood flow reduction, there is marked impairment in neuronal energy metabolism, astrocytosis, reduced protein synthesis, an increased amount of protein abnormalities and oxidative stress, spatial memory loss, endothelial cell damage, as well as Aβ1-42 upregulation and brain atrophy with ultimate cellular death [102]. Regarding human studies, there are several important points that give weight to the vascular hypothesis of AD. The significance of vascular risk factors has been confirmed by epidemiological data [104,105]. Individuals with MCI and a risk for development of AD have cerebral hypoperfusion and hypometabolism as shown by simple-photon emission computed tomography (SPECT) and PET-scan studies [106,107,108,109]. Antihypertensive therapy has been demonstrated to reduce AD risk [110,111]. Microvascular changes in the brain have been discussed as important factors in AD development in both the clinical and pathological context [110,111].

Cerebral vascular perfusion is also under control of circadian system. Conroy et al. studied the daily rhythm of cerebral blood flow velocity (CBFV) in humans over a 30 h period of sustained wakefulness in order to determine if time-of-day differences in perfusion can be explained solely by behavioral changes and metabolic needs, or if an endogenous rhythm independent of sleep is present [112]. Results suggest that human CBFV clearly follows an endogenous circadian rhythm worth of further exploration in context of cognitive performance decrements and cardiovascular/cerebrovascular events. Similar findings were reported in rats by laser-Doppler flowmetry [113]. There seems to be a circadian periodicity in cerebral blood flow independent of circadian changes in blood pressure and locomotor activity. Circadian rhythm influence on perfusion and brain metabolism should be considered in future research of vascular function importance in the etiopathogenesis of AD.

### 2.6. Metabolic Dysfunction

Recently, metabolic dysfunction has gained more attention in AD research given its association with neurodegenerative changes reported both in the clinical and experimental setting [114]. Metabolic abnormalities seem to take place early in the course of the disease, indicating pathophysiological processes affecting energy systems could be important in etiopathogenesis of AD. This is clinically important because abnormalities in glucose metabolism can be assessed in vivo using fluorodeoxyglucose positron-emission tomography (FDG-PET). An increasing amount of evidence suggests FDG-PET could be valuable in assessment of early AD [115,116,117]. Growing recognition of insulin as one of the salient regulators of brain metabolism has led to the hypothesis that disrupted insulin signaling might be one of the key processes in the development of the disease [118,119,120]. Although the brain was long considered to be insulin insensitive, evidence accumulated over the last few decades suggests pleiotropic effects of insulin regulate some of the processes of utmost importance for its homeostasis such as glucose metabolism, synaptic plasticity, and neuronal growth or survival [120].

Metabolic processes exhibit pronounced circadian variation at various levels. The literature suggests that this variation, expected in the context of polarized behavioral pattern consisted of resting and fasting versus activity and feeding, is influenced both by external and internal mechanisms [121]. Importance of circadian regulation of metabolism is evident from research suggesting exogenous [122,123] and endogenous [124,125] disruptions of circadian rhythm can induce changes related to metabolic syndrome both in human and animals. In context of epidemiological evidence highlighting metabolic dysfunction as an important risk factor for developing AD [126,127,128], supplemented by animal studies proposing pathophysiological causative mechanisms of underlying metabolic disruption in neurodegeneration [129,130], circadian-related metabolic abnormalities should be considered as a potential factor in the development of age-related neuropathology. 

### 2.7. Melatonin

The circadian pattern of circulating melatonin, the most popular biological chronomodulator, is regulated by light signals that modulate SCN activity through retinohypothalamic tract projections beginning in the photoreceptive retinal ganglion cells [131]. It has been shown that nocturnal exposure to light can acutely suppress melatonin synthesis through degradation of pineal N-acetyl transferase [132], the key regulator of melatonin production. Although melatonin is best known for its chronobiological role, it is involved in diverse physiological functions, as is the case with many other evolutionary conserved molecules. Regarding its chronobiological activity, melatonin fulfills a dual role. It communicates circadian signal from the SCN ‘zeitgeber’ to the peripheral cells, but also regulates SCN activity in a feedback manner [133]. In order for any biological system to work properly, synchrony at any organizational level is essential. Failure to preserve harmonization dramatically reduces the amount of allostatic load that can be tolerated by the system and consequently increases susceptibility to develop disease. Loss of melatonin circadian pattern due to melatonin axis dysregulation or due to circadian disruption could lead to asynchrony and impair homeostatic mechanisms.

Regarding its non-circadian function, melatonin seems to be important for immune system function, cell protection, mitochondrial function and biogenesis, and energy homeostasis. Melatonin has been regarded as an immunological buffer due to its immunostimulant properties under basal or immunosuppressive conditions and its ability to suppress immune response and inflammation when immune reaction is excessive [134]. Melatonin itself acts as a free radical scavenger. It has been shown that a single molecule of melatonin can generate products that can collectively eliminate 10 free radicals [133]. Moreover, melatonin upregulates several important antioxidant enzymes [135], down-regulates pro-oxidant enzymes, and induces “radical avoidance” by decreasing mitochondrial electron leakage [136]. Melatonin also increases ATP generation and increases mitochondrial biogenesis, as well as mitophagy, thereby supporting functional cellular energetics [137,138]. Additionally, melatonin could ameliorate insulin resistance and hyperglycemia, as well as inhibit blood brain barrier disruption-pathophysiological events considered to be of importance in the development of AD [139]. In patients affected by AD, melatonin levels are decreased and melatonin circadian rhythm is dysregulated [140]. This has even been noted in the preclinical stages of the disease [140].

Numerous other hypotheses that have been generated in order to find a singular cause of AD (e.g., the metal ion homeostasis hypothesis or the cholesterol hypothesis [56]), might elucidate other potential pathophysiological mechanisms mediating circadian-driven AD pathogenesis [141]. Given the extremely complex pathophysiology of the disease and multiple genetic and environmental factors proven as risk modifiers and drivers of the disease in different contexts, it could be more likely that there are multiple subtypes of the disease, with every factor representing a burden of different significance in each individual patient. Circadian rhythm is related to many different processes involved with AD pathogenesis and should be further examined in this context.

## 3. Circadian Rhythm, Sleep, and Alzheimer’s Disease from Clinical Perspective

Circadian alterations occur both during healthy aging and in age-related diseases such as AD. However, some data suggest circadian rhythm disruption in AD is more pronounced and could be a useful additional indicator of the disease development. Some of the changes that seem to occur are related to sleep and involve nocturnal sleep fragmentation, increased wakefulness, and decreased levels of daytime activity with diurnal napping [19,142,143]. Although additional research is needed to fully understand the diagnostic significance of circadian disruption in preclinical AD, it would be beneficial to include sleep pattern related questions in medical history examination. These sleep disturbances have a significant impact on patients and their caregivers, and present a major risk factor for early institutionalization.

Specific sleep alterations include loss of slow-wave sleep (SWS) and REM sleep. Some studies suggest that the REM phase stays unaffected during the early stages of the disease, but starts decreasing in later stages [143]. Slow-wave sleep, which represents stage three of non-REM (NREM) sleep and is often called deep sleep, has also shown decreased duration in patients affected with AD [144]. Electroencephalograms (EEGs) of patients with AD, compared to healthy controls and patients suffering from MCI, display a decreased density of K-complexes (KC), which are one of the hallmarks of NREM sleep [145]. It should also be noted that KC density was in positive correlation with Mini Mental State Examination (MMSE) scores, acknowledging the fact that sleep disruption parallels severity of dementia. It has been long known that sleep has a restorative function in the brain and is involved with memory retention [142]. Slow-wave sleep specifically has proven to be especially significant for memory retention [142]. The mechanism behind this phenomenon has been proposed by Tononi and Cirelli in 2006 [146]—Slow waves found in EEG stand for lower neuronal energy, which is more sustainable and favorable for synaptic plasticity and memory consolidation. Elderly people are known to have decreased SWS, a characteristic which is even more emphasized in AD, further aggravating already existing memory problems. Other circadian changes were also observed in AD patients. Most et al. reported that AD patients have higher proximal skin temperature in comparison with age-matched controls [147]. Volicer et al. observed AD patients have higher amplitude of the fitted cosine temperature curve, and later temperature acrophase (time of peak) than the healthy subjects did [148]. In AD and sleep deprived patients, SWS is diminished, meaning neurons spend less time in a hyperpolarized state and more in energized states, producing even more Aβ protein. A bidirectional link between these two exists, although it is still unclear which one holds the causative role [33]. 

Alzheimer’s disease patients experienced less diurnal motor activity, a higher percentage of nocturnal activity, lower inter-daily stability of motor activity, and a later activity acrophase than did the healthy individuals [148]. Moreover, higher levels of regular nocturnal motor activity are in correlation with aggressive behavior, agitation and restlessness occurring during the late afternoon or early evening often referred to as sundowning and diagnosed in 13–66% of patients [19]. Circadian disruption presenting as excessive night-time activity and wakefulness also seems to be an important risk factor for early institutionalization [149].

## 4. Alzheimer’s Disease and Circadian Disruption—A Positive Feedback Loop

Although most of the research is focused on the causative role of circadian disruption in AD, pathophysiological changes that occur during the disease development often induce or encourage circadian misalignment. Schmitt et al. have shown that Aβ can disturb molecular clock and induce changes to metabolic circadian rhythmicity and cellular bioenergetics [150]. Considering the importance of mitochondrial bioenergetics in AD development [151] and relevance of circadian metabolism in health and disease [152], Aβ should be taken into account as both the potential cause and consequence of circadian allostasis. Moreover, pathological tau physiology induces circadian changes at both a molecular and behavioral level, as indicated by the research on the mouse model of taupathy Tg4510. Taupathy in SCN was observed in parallel with impaired core clock protein PER2 cyclic expression in the hypothalamus. A similar observation was made for the key circadian proteins PER2 and BMAL1 in the hippocampus [153]. Inflammation, discussed earlier as a potential causative factor in AD pathogenesis, is also known as a circadian modulator. Lipopolysaccharide (LPS), often used in research to generate an inflammatory response, induces phase shift through generation of proinflammatory cytokines and their effect on SCN [154]. Oxidative stress, another significant etiopathogenetic factor in AD and recognized driver of disease progression, can entrain circadian clock systems as proven by both in vitro and in vivo studies [155]. Although peripheral circadian clock shift by oxidative stress is discussed in context of adaptation to stress, chronic dyssynchrony of molecular clocks can undermine homeostasis and health. 

**Figure 1:**
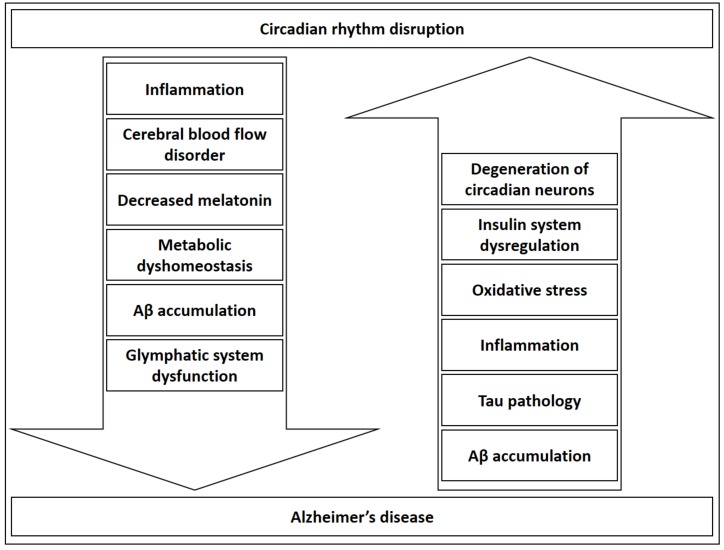
Circadian rhythm regulates some of the key processes thought to be involved in Alzheimer’s disease etiopathogenesis and progression such as inflammation, cerebral blood flow, melatonin production, amyloid β clearance, glymphatic system and metabolism. Moreover, some of the events associated either with early pathobiological changes in Alzheimer’s disease, or late pathophysiological consequences are known as potential circadian disruptors. The most important AD-related circadian disrupting mechanisms include neurodegeneration of neurons and neural pathways critical for circadian signaling, insulin system dysregulation, oxidative stress, inflammation, and pathological homeostasis of Aβ and tau. Aβ—amyloid β.

The insulin system, which is recently even discussed in the context of AD [118,120,156], also modulates the circadian rhythm. Insulin resets the hepatic circadian metabolic clock in order to adjust peripheral rhythms to nutrients and the endocrine environment [157], and regulates central and peripheral clock sensitivity and function [158,159]. It can be postulated that metabolic dysregulation might induce and further accentuate AD development and, in parallel, circadian rhythm dyshomeostasis. This could be true not only for insulin, but for the metabolism in general, in light of findings that reveal a bi-directional regulation of metabolic processes and circadian rhythm reviewed by Bailey et al. [121]. Furthermore, circadian system failure could be a result of degeneration of neurons regulating the clock [160,161] providing another possible explanation for evident circadian dysregulation in AD. As evident from possible circadian consequences of AD and possible circadian-related mechanisms in AD etiopathogenesis (see Section 2. Shared Pathogenic Mechanisms of AD and Circadian Rhythm Dysfunction), circadian rhythm dysregulation can be discussed as both a disease driver and potentiator recognized in a clinical context (Figure 1).

## 5. Diagnostic Possibilities

The bi-directional relationship between sleep disorders and Alzheimer’s disease gives added importance to the task of efficiently and accurately identifying sleep pathology and/or circadian rhythm dysfunction. This is relevant in patients already diagnosed with AD, whose cognitive deterioration may be aggravated by sleep disturbances; as well as in at-risk populations, where changes in sleep patterns and irregularities in rest-activity rhythms can potentially be used as an early diagnostic marker for AD, as the onset of these symptoms can precede clinical diagnosis of the disease by several years [162]. In patients with dementia, abnormal rest-activity timing has demonstrated to be a predictor of institutionalization and is even associated with decreased survival [149,163].

Human circadian rhythms can be directly observed using several methods, such as measuring plasma melatonin levels (which normally peak around 2 h before awakening) or tracking core body temperature using rectal thermometry (with a low 1–2 h before awakening, inversely to melatonin levels) [164]. Rectal thermometry is more commonly used due to its greater availability, but has a disadvantage over plasma melatonin measurement in that temperature levels are vulnerable to both internal and external confounding factors. Melatonin levels also have a higher amplitude and distinct phases [165,166]. While these methods have proven invaluable in expanding our understanding of circadian rhythms in an experimental setting, they are perhaps too invasive for routine use in clinical practice. Instead, the behavioral output of the circadian rhythm—mainly the sleep–wake cycle and activity levels—can be used as an easily observable, yet relevant alternative parameter [167]. It should be noted that, while ‘sleep–wake cycle’ is often used synonymously with ‘circadian rhythm’, sleep timing is not a perfect reflection of the circadian rhythm as it is also dependent on other factors, such as sleep pressure and arousal [33]. 

Reports of ‘sleep–wake disturbances’ are often nonspecific, encompassing anything from decreased sleep time, delayed sleep, frequent awakenings or daytime sleepiness to confusion, wandering or aggression occurring in the evening [168]. The nature of these issues is multifold, the degree of their coexistence not well documented. Some may represent distinct symptoms, tied to distinct pathophysiological mechanisms. It appears, for example, that ‘sundowning’, a hallmark behavioral symptom in AD consisting of agitation in the late afternoon to early evening, which has been associated with objective abnormalities in circadian rhythm, does not necessarily correlate with complaints of ‘sleep disturbances’ [148]. However, sufficiently analyzed and described methods can provide valuable data regarding disease progression even at an early stage of the disease. In a recently published work, decreased sleep efficacy in 45–75 year old healthy individuals monitored by actigraphy was indicative of amyloid deposition, although their cognitive function was normal [169]. 

### 5.1. History and Questionnaires

The first step in the assessment of sleep–wake disturbances is as precise a history as possible, which, in AD patients, usually entails a collateral history given by a caregiver. In addition to information about comorbidities, medication, and habits relevant to sleep hygiene, this should ideally include a sleep journal, detailing the timing and quality of nighttime sleep, any nighttime awakenings, the nature and frequency of nighttime behavioral symptoms and the frequency and duration of daytime naps. Traditional sleep questionnaires such as the Pittsburgh Sleep Quality Index or the Epworth Sleepiness Scale seem to be of limited use, as AD patients have been shown to underscore themselves in early stages of the disease, while caretakers are likely to affect the score by helping patients complete the questionnaires in later stages [170,171]. Tools such as the Neuropsychiatric Inventory (NPI) or the Sleep Disorders Inventory (SDI) may be useful for quantifying sleep disturbances in AD. The SDI contains questions regarding seven sleep symptoms common in AD, rating them for severity, frequency, and caregiver distress. It shows excellent correlation to actigraphy findings, is short, relatively simple, and (unlike traditional sleep logs) requires only one visit to be administered [172]. The history should also screen for comorbid primary sleep disorders with questions regarding snoring, leg discomfort, witnessed apneas or leg movements during sleep. Primary sleep disorders are more prevalent in AD compared to the healthy aging population. Sleep-related breathing disorders such as obstructive sleep apnea (OSA), a common cause of nighttime awakenings and daytime sleepiness, can be found in 40–70% of AD patients [173,174]. Restless legs syndrome (RLS), which primarily leads to difficulties in falling asleep, is thought to be present in as much as 24% of patients with dementia [175]. Determining if symptoms can be attributed to these disorders is relevant, since both OSA and RLS are potentially treatable.

### 5.2. Polysomnography

Polysomnography (PSG) is the gold standard test for the objective assessment of sleep. Polysomnography can provide information on parameters ranging from sleep latency, duration, and architecture to heart rate, oxygen saturation, respiratory movements, limb movements, and position switches. It is virtually indispensable for the diagnosis of sleep-related breathing disorders. However, objectively determining circadian rhythm disorders and other AD-related sleep disturbances requires a patient to be observed over seven days or more, making the expensive and equipment-heavy PSG a rather impractical diagnostic method to use [176]. Additionally, the multitude of sensors and the sleep lab environment in general can be quite uncomfortable and disruptive to sleep, requiring a certain degree of cooperation from subjects, which can be problematic in patients with dementia.

### 5.3. Actigraphy

Another tool for objective sleep analysis, especially useful in examining circadian rhythm disturbances, is actigraphy. Actigraphy involves continuously wearing a wrist watch-like device which monitors bodily movement for a designated period of time (ideally at least one week). It provides information on periods of activity and inactivity, which are assumed to correspond to periods of sleep and wakefulness. Studies repeatedly show good correlation of actigraphy-based assessments with EEG findings in both healthy subjects and dementia patients, especially in determining total sleep time [176,177,178]. The test is less costly than PSG, as well as considerably less intrusive, making it easier for patients with dementia to tolerate. However, the strength of actigraphy in identifying variables such as daytime naps and sleep latency is limited [179]. Furthermore, its specificity in discerning can be reduced in hospitalized or bedridden patients, who comprise a significant portion of the AD population. The American Academy of Sleep Medicine recommends for actigraphy to be used concurrently with sleep journals to provide contextual information and minimize the influence of confounding factors [176]. Some actigraphs also contain light sensors to help identify the major sleep period and any nighttime awakenings.

## 6. Potential Therapeutic and Preventive Strategies

Diagnostic strategies focused at circadian disruption in AD could help us design a plan for prevention and treatment of circadian dyssynchrony in order to slow down disease progression and alleviate some of the symptoms. More research is needed to generate evidence based guidelines backed up by strong scientific evidence, but current knowledge on the subject offers some potential strategies discussed in the following paragraphs.

### 6.1. Optimizing Medication and Comorbidities

Management of circadian and sleep–wake disorders associated with AD should begin with managing any comorbidities which could interfere with sleep. Prostatic hypertrophy, fecal impaction, congestive heart failure, chronic obstructive pulmonary disease, gastroesophageal reflux, and arthritis are all significant causes of pain and/or discomfort common in the elderly which need to be addressed before proceeding with any sleep-related interventions [180]. The same is true for psychiatric illnesses which affect sleep, such as depression, which is estimated to be present in up to 50% of AD patients [181]. Additionally, dosage of any medication that can cause either sleep latency, frequent awakenings or daytime sleepiness should be reviewed. Examples include diuretics, bronchodilators, corticosteroids, antihistamines, antidepressants, and anti-Parkinsonian drugs [182]. Acetylcholinesterase inhibitors donepezil and rivastigmine, used in the treatment of AD itself, have been known to cause insomnia and nightmares when taken at night; however, when dosage is moved earlier in the day, they appear to improve sleep quality and increase REM sleep percentage [183,184]. The recently FDA-approved suvorexant, an orexin receptor antagonist which has proven to increase sleep efficiency, might hold promise [185].

### 6.2. Sleep Hygiene and Environmental Measures

The term ‘sleep hygiene’ refers to a set of behavioral and environmental recommendations intended to improve sleep quality. The basic principles of sleep hygiene include keeping a regular sleep schedule, engaging in daily exercise, minimizing daytime naps, restricting the use of caffeine, alcohol and tobacco, keeping the sleep environment dark and quiet, and generally restricting light close to bedtime [186]. Often included stimulus-control therapy strategies include developing a fixed bedtime routine or using the bed for sleep only.

Behavioral and environmental factors likely contribute to changes in sleep patterns that occur with age. The daily schedules of older adults often lose structure after retirement, mealtimes become irregular, pain and other comorbidities lead to less physical activity, while boredom and loneliness become more prevalent [187]. Patients with dementia often spend excessive time in bed, with frequent daytime naps. This is sometimes even encouraged by caregivers in order to ease burden [188]. Sleep hygiene is especially poor in elderly living in nursing homes. Nursing home residents are exposed to only around 9–11 min of bright light a day (compared to around 60 min for young adults and 40 min for elderly living at home), with the average plummeting to as low as 1 min a day for severely demented institutionalized patients [166,189,190]. The nursing home environment involves a higher than ideal level of noise and light during the night, as well as frequent interruptions by staff (for example, in order to do incontinence care or check for pressure sores) which account for a significant portion of nighttime awakenings [191]. Improving these determinants is, at first glance, a good early target for intervention.

Sleep hygiene education programs for patients with dementia and their caregivers have had modest success, but require a high degree of caregiver involvement and are perhaps less feasible in institutionalized settings [192,193]. The outcomes of interventions attempting to improve sleep quality by increasing daytime physical and/or social activity have been mixed [194,195,196]. Environmental interventions alone for nursing home patients (such as reducing noise levels during wetness checks or turning off unwatched television sets) have had similarly limited success, with significant improvements in noise and light levels producing a minimal improvement in sleep and agitation scores [197]. The most promising results have been yielded by multicomponent interventions combining several of these approaches [198,199,200]. While evidence for the effectiveness of these non-light based, non-pharmacological strategies remains inconclusive, this is to a large extent a matter of methodological weaknesses in the available studies and should not discourage from further implementation of these methods [201]. They remain the basis for treatment of sleep–wake disturbances in dementia patients due to their simplicity and lack of adverse effect.

Artificial light is another important potential circadian disruptor that should be addressed. About 75% of the world population is exposed to artificial light during the night [202]. A significant amount of nighttime light exposure is due to electronic devices. Some of these devices emit monochromatic blue light that activates intrinsically photosensitive retinal ganglion cells [203]. Even low levels of artificial light before bedtime from an e-book reader can reduce melatonin secretion, reduce sleepiness, increase time to fall asleep, and reduce next morning alertness [204].

Noise, defined as an unwanted sound, affects people both psychologically and physiologically. It has been reported that excessive noise can suppress the immune system and increase chances for an infection, increase gastric secretion, stimulate pituitary and adrenal gland, and decrease fertility [205]. Noise can also induce circadian rhythm disruption, likely indirectly through sleep quality reduction [205,206,207]. Noise has been associated with Alzheimer’s disease. It has been proposed that chronic noise exposure can induce tau hyperphosphorylation, Aβ accumulation, NFT formation, and induce excitotoxicity and oxidative stress [208]. Chronic noise has also been shown to disrupt glucose homeostasis and induce insulin resistance, increase corticosterone and induce gut microbiota dysbiosis, and increase inflammation [209,210]. This is why the World Health Organization recommended that noise levels inside hospital wards should not exceed 30 dBA at night. Unfortunately, studies report that noise levels inside hospitals are much higher [205].

In order to slow down AD progression and reduce the burden of circadian desynchronization, AD patients should reside in an optimal environment without circadian disruptors. To assure this, high enough levels of light should be distributed throughout the day and low enough levels throughout the night, while environmental noise should ideally be reduced during the night. If possible, electronic device arrangements should follow aforementioned concepts.

### 6.3. Physical Activity

Scheduled exercise has been proposed as one of the measures for prevention of circadian disruption and stimulation of circadian resynchronization. The mechanism through which it achieves its effects is still unclear, with many methodological obstacles exist in both basic and applied research on the topic [211]. However, given the possibility that exercise can be used to potentiate other circadian resynchronization methods [212] combined with evidence that leisure-time physical activity is protective against AD [213], appropriate physical activity recommendations should be settled for patients with different stages of the disease and for healthy people with risk factors for AD development.

### 6.4. Meal Timing

Circadian disruption by forced activity during the sleep phase changes meal timing in rodents [214]. Desynchronization of the circadian rhythm with consequences on meal timing and content (in favor of carbohydrates) has also been shown in night-shift workers [214,215]. Interestingly, it has been shown that meal timing, quality, and quantity can prevent circadian disruption and even induce circadian resynchronization. In animals, hypocaloric diets and consequent body weight reduction enhance circadian amplitude regardless of the circadian phase in which food was restricted [214,216]. Evidence indicate food timing can be a powerful synchronizing [217] or desynchronizing [218] for the circadian system exerting its activity through complex interplay of central and peripheral oscillators [219]. It has been suggested resynchronizing peripheral and central oscillators by food could also be beneficial in AD [220], although high quality clinical data is needed to formulate this hypothesis into evidence driven recommendation. Because metabolic and digestive temporal adjustments to meal time are important for proper nutrient extraction from food with consequent metabolic, endocrine, and other physiological changes [221], resynchronization of disrupted circadian rhythm and proper nutrient timing may be beneficial for AD patients. One study suggested that cognitive and behavioral deterioration and circadian changes make traditional meal practices, with the most energy-dense meals being either lunch or dinner, inappropriate for AD patients [222]. Food delivery programs should be redesigned to provide the most energy-dense high-quality food in the morning when peak energy consumption occurs in AD patients [222].

### 6.5. Bright-Light Therapy

A study from 2000 [223] showed that 2 h of bright-light therapy (BLT, around 3000 lux) administered daily during the course of 4 weeks improved the circadian rhythm of 27 AD patients (mean age was 79.9 years). Specifically, it decreased daytime sleep and increased nighttime sleep. Clinical dementia rating (CDR) scores remained the same throughout the study, but MMSE scores improved in all patients. In conclusion, BLT resulted in significantly better sleep–wake cycles and even improved the cognitive state in people diagnosed with early stages of AD. Some studies, however, found no positive effects of light therapy [224], while another study [225], which included 70 institutionalized AD patients, found no difference in sleep–wake cycles between the control group and the group that received BLT of about 2500 lux for 1 h in the morning during eleven weeks. However, the intervention group has proven to gain a stability in their rest-activity rhythms. A probable reason for the discrepancy of these results lies in differences in the mean age of participants, number of participants, duration of increased light exposure, and strength of the light used. Nevertheless, some research [223] stands out with highly positive results, prompting for conclusive RCTs with a larger number of participants.

### 6.6. Melatonin

Melatonin was proposed as a potential treatment of circadian-related problems in AD. Although its effects were considered promising based on pathophysiological data, empirical clinical evidence so far does not support its effectiveness in manifest AD. However, there are indications it could improve sundowning [226,227]. Though, other studies [228,229], have shown no benefits of melatonin for sleep quality or decrease in agitation. Possible explanations for these negative results are short duration of trials and the inclusion of patients with varying stages of AD and dementia. Long-term studies [227,230] have proven amelioration of sleep disorders in AD patients.

Add-on prolonged release melatonin also showed increased sleep efficiency and higher cognitive performance results, as measured by MMSE [231]. Finally, a meta-analysis of randomized control trials on melatonin as monotherapy was done [232]. It showed improved total sleep time in AD patients, but had no effect on their cognitive states. It is worth noting that this meta-analysis took into consideration studies of both short and long duration (from 10 days to 24 weeks. For a more thorough review of evidence on melatonin in context of AD please see Cardinali et al. [233]).

Because of its intriguing biological effects (immunoregulatory, cytoprotective, promoting mitochondrial function, biogenesis and energy homeostasis), melatonin is still an interesting candidate worth exploring in this context. It is possible that advancement in research of circadian rhythms and more robust studies focused on route and time of administration will elucidate some of the discrepancies in melatonin effectiveness between animal research and the clinical setting.

### 6.7. Combined Bright Light Therapy and Melatonin

A study from 2008 [234] found that bright light therapy alone did not improve nighttime sleep or rest activity rhythm, but did so when used together with melatonin. A randomized controlled trial included fifty subjects with AD and mean age of 86 years. One group was exposed to 1 h of BLT (>2500 lux in gaze direction) in the morning for 10 weeks and was given a placebo. The second group was exposed to the same light treatment, but was additionally given melatonin (5 mg, a moderate pharmacological dose) in the evening, while a third group received typical indoor light of about 150–200 lux. The second group of subjects experienced more daytime activity and less daytime somnolence, resulting in a healthier diurnal pattern. However, no noticeable changes were observed regarding nighttime sleep. It remains unclear whether these improvements were caused solely by melatonin or melatonin’s synergy with bright light treatment.

## 7. Conclusions

This article uncovers a complex network of interactions, scoping between circadian rhythm disturbances and AD pathogenesis. The narrative form of this review should ideally demonstrate these complexities in an insightful manner as a resource that evaluates the most up-to-date knowledge related to the topics. The primary value of such an article is the possibility to address specific findings in a more comprehensive manner, without certain methodological restrictions that are objectively defined in systematic reviews. However, the limitations of this article stem from its subjectivity and degree of freedom, which contributes to possible biases in terms of mode of presentation and references chosen. In respect, the authors aspired to maintain an academic approach by primarily, basing claims in scientific evidence in a manner that would be deemed appropriate for a narrative review article.

To conclude, a growing body of evidence (both from clinical studies and animal research) supports a complex and meaningful relationship between circadian rhythms and Alzheimer’s disease. Although numerous hypotheses have been generated in order to explain the etiology of AD, it still remains to be fully elucidated. However, circadian rhythm interacts with most if not all systems and risk factors known to be responsible for AD development and progression. Therefore, it represents an interesting target for possible disease prevention and treatment. The relationship between AD and circadian rhythm seems to be bi-directional and the optimum goal would be to meaningfully influence disease progression by circadian intervention, or at least provide valuable symptomatic relief and greatly reduce socioeconomic costs and suffering. In the era of precision medicine, which strives toward fully personalized care, a deeper understanding of the circadian rhythm–AD relationship should help provide a successful evidence-based comprehensive approach and more effective treatments.
